# Phantom-based performance comparison of two commercial deep learning CT reconstruction algorithms with super- and normal-resolution settings

**DOI:** 10.1186/s41747-025-00670-2

**Published:** 2026-01-26

**Authors:** Joël Greffier, Catherine Roy, Djamel Dabli, Jean-Paul Beregi, Maxime Pastor

**Affiliations:** 1 IMAGINE UR UM 103, Montpellier University, Department of Medical Imaging, Nîmes University Hospital, Nîmes, France; 2 Diagnostic Imagery Department, Nouvel Hôpital Civil (NHC), Hôpitaux Universitaires de Strasbourg, Strasbourg, France

**Keywords:** Artificial intelligence, Deep learning, Image enhancement, Image processing (computer-assisted), Multidetector computed tomography

## Abstract

**Objective:**

We compared a super-resolution deep learning image reconstruction (SR-DLR) algorithm with a normal-resolution (NR)-DLR algorithm according to radiation dose for abdominal computed tomography (CT).

**Materials and methods:**

An image-quality phantom was scanned with an energy-integrating detectors CT unit at three volume CT dose index radiation dose levels (12.7, 5.9, and 3 mGy). Images were reconstructed using a 1,024^2^ matrix for SR-DLR and a 512^2^ matrix for NR-DLR, for three DLR levels (level-1, level-2, and level-3). Noise power spectrum (NPS) and task-based transfer function (TTF) for iodine and Solid Water^®^ inserts were computed; TTF values at 50% (f_50_, mm^-1^) were used to quantify spatial resolution. The detectability index (d’) was computed for two simulated lesions.

**Results:**

Noise magnitude values were lower with SR-DLR than with NR-DLR for level-2 (-27.6 ± 3.8%) and level-3 (-43.5 ± 1.4%), the opposite for level-1. Average NPS spatial frequency was higher with SR-DLR than with NR-DLR for all radiation dose levels for level-1 (55.9 ± 16.7%) and level-2 (20.1 ± 13.9%) and the opposite for level-3, except at 12.7 mGy. For both inserts, f_50_ was higher with SR-DLR than with NR-DLR at each radiation dose and DLR level. For simulated lesions and all DLR levels, d’ values were higher with SR-DLR than with NR-DLR (level-1, 6.0 ± 2.0%; level-2, 45.7 ± 5.0%; level-3, 75.2 ± 7.3%).

**Conclusion:**

Compared to NR-DLR, SR-DLR improved spatial resolution and detectability of simulated abdominal lesions; image noise was reduced with SR-DLR only for level-2 and level-3, while image texture was better for level-1 and level-2.

**Relevance statement:**

Super-resolution DLR with a 1,024^2^ matrix size improved spatial resolution and detectability of simulated abdominal lesions compared to normal-resolution DLR. Validation in clinical settings is necessary before translation into routine practice.

**Key Points:**

- The performance of a new deep learning super-resolution image reconstruction algorithm (SR-DLR) was compared to a normal-resolution (NR)-DLR algorithm using an image-quality phantom for an abdominal energy-integrating detector CT protocol.
- SR-DLR with a 1,024^2^ matrix improved spatial resolution and detectability of simulated abdominal lesions compared to NR-DLR with a 512^2^ matrix.
- Using SR-DLR, therefore, presents numerous prospects for improving abdominal CT images and a high potential for reducing the radiation doses.

**Graphical Abstract:**

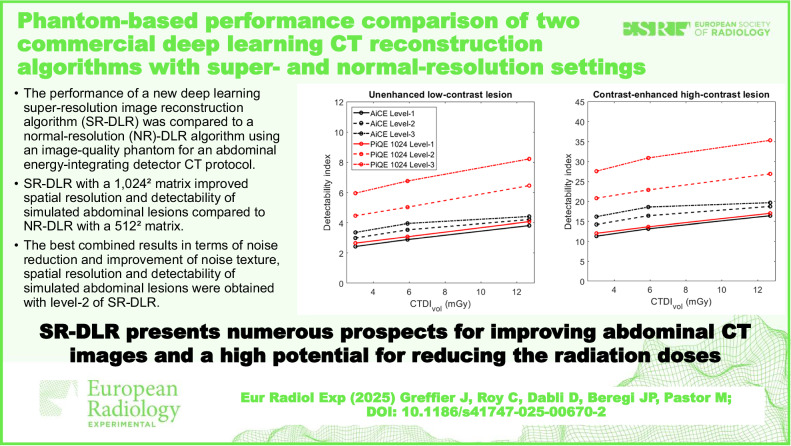

## Background

Recently, with the development of artificial intelligence, deep learning reconstruction (DLR) algorithms are being used in clinical routine for computed tomography (CT) examinations [1–5]. These DLR algorithms have been developed to continue improving image quality, especially by reducing image noise, and to compensate for one of the limitations of iterative reconstruction algorithms, which change image texture (*e.g*., image smoothing, coarser granularity, unnatural images) [1, 2, 6]. In conventional CT, most DLR algorithms feature a convolutional or deep neural network to differentiate the signal from the noise [7–9]. With DLR algorithms, neural networks are trained using high-quality CT images from a large database of patients and/or phantoms [7–9]. In abdominal CT, using DLR algorithms has allowed to improve the quality and diagnostic confidence of images and/or to reduce the radiation doses delivered to patients [10–20].

In 2021, a new DLR algorithm dedicated to cardiac image reconstruction was developed [21, 22]. This algorithm, called Precise IQ Engine (PIQE, Canon Medical Systems), features a three-dimensional deep convolutional neural network trained with cardiac image data acquired on an ultra-high resolution CT system (Aquilion Precision, Canon Medical Systems) with 0.25-mm detector elements and reconstructed with the DLR algorithm AiCE (Advanced intelligent Clear-IQ Engine, Canon Medical Systems) and a matrix size of 1,024^2^ pixels [23]. Originally, PIQE was only available on the Aquilion One Prism CT system, and images could only be reconstructed with two reconstructed image thicknesses (0.5 and 1 mm) and one matrix size of 512^2^ pixels. Many studies on phantoms and in clinical routine have shown that, compared to the DLR algorithm used until now, the first version of PIQE improves spatial resolution and low-contrast detectability, giving a sharper image, whilst reducing image noise [22, 24–26].

In 2023, with the development of a new CT system (Aquilion One INSIGHT Edition), two new kernels (Body and Lung) and a matrix size of 1,024^2^ pixels are now available with PIQE. This has been made possible by the expansion of the datasets used for training the PIQE convolutional neural network – initially based solely on cardiac images—and also thanks to the performance of this new CT scanner, which allows this extended matrix to be used in clinical routine. Routine clinical use of this extended pixel matrix has opened many possibilities to maximize the spatial resolution not only of cardiac CT images but also of other clinical applications with the two new kernels. The advantages of PIQE in super-resolution (SR)-DLR mode with this new extended matrix in comparison with AiCE in normal-resolution (NR)-DLR have already been demonstrated in cardiac imaging [23].

PIQE could also help to improve the quality of abdominal CT images, where low-contrast structures strongly depend on image noise and spatial resolution conditions. To the best of our knowledge, only two studies have been published and compared PIQE as an SR-DLR with AiCE as an NR-DLR on patients [27] or phantoms [28] for abdominal CT protocols. However, neither study conducted a comprehensive task-based image quality assessment nor examined the impact of SR-DLR on the detectability of simulated abdominal lesions.

Thus, the purpose of this study was to compare the image quality performance of two commercial deep-learning reconstruction algorithms (SR-DLR, PIQE and NR-DLR, AiCE) using a standard phantom scanned with an abdominal CT protocol and evaluated through task-based image quality metrics.

## Materials and methods

### Phantom

**Fig. 1:**
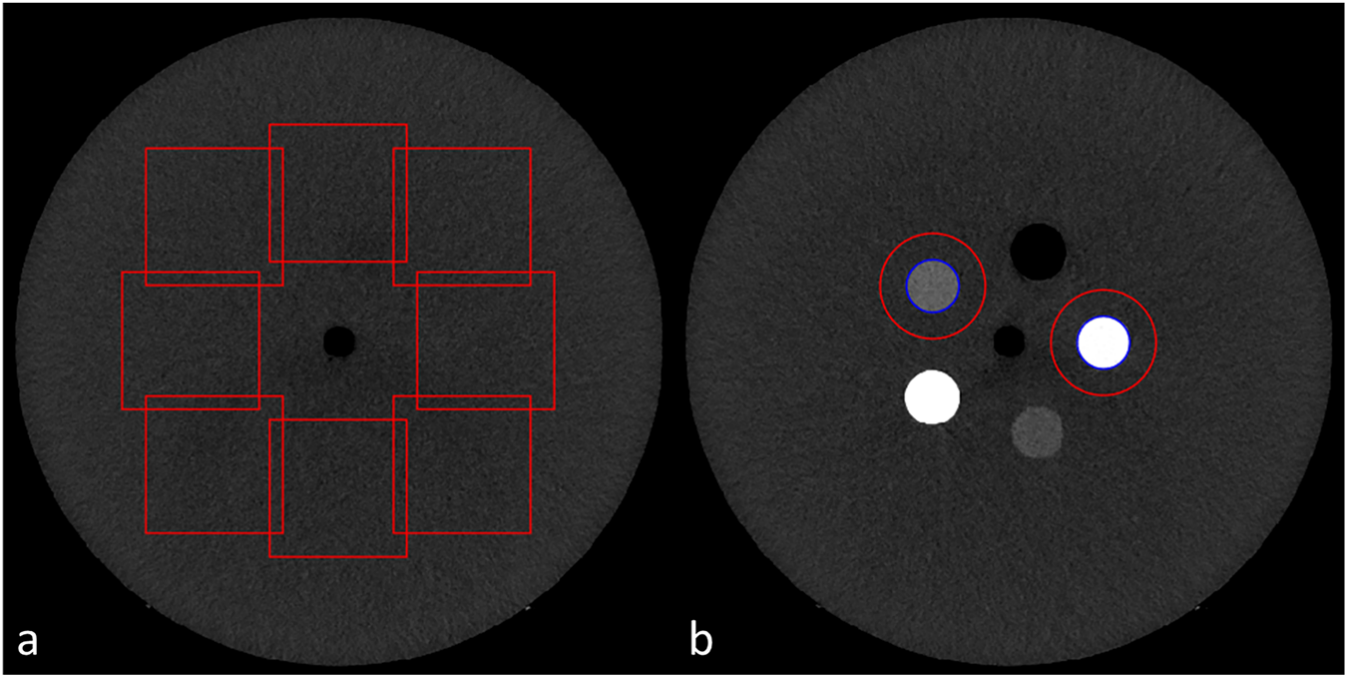
31 cm diameter section of the Mercury 4.0 phantom used in this study. **a** Eight square regions of interest (ROIs) were used to assess the noise power spectrum (NPS). **b** Two circular ROI were used to compute the task-based transfer function (TTF) on Solid Water^®^ and 10 mg/mL iodine inserts

For this study, the 31-cm diameter section (body mass index 27 kg/m²) of the Mercury v.4.0 image quality phantom (Gammex) was used [29]. This phantom (52 cm in length) is composed of 5 sections of 5 different diameters: 16, 21, 26, 31, and 36 cm. Each section consists of a homogeneous part of polyethylene (phantom’s background material) and a second part consisting of 4 inserts (Solid Water^®^ HE (Gammex), Bone Mimicking Material, polystyrene and iodine at 10 mg/mL) and an air hole with a diameter of 2.5 cm placed in the background phantom’s background material. In this study, the homogeneous part of this section was used to compute the noise power spectrum (NPS) (Fig. 1a) and the second part was used to calculate the task-based transfer function (TTF) on the Solid Water^®^ and iodine at 10 mg/mL inserts (Fig. 1b).

### Acquisition and reconstruction parameters

Acquisitions were performed on the Aquilion One INSIGHT Edition (Canon Medical Systems) equipped with AiCE and PIQE. All CT acquisitions were made with a tube voltage of 120 kVp, a rotation time of 0.5 s/rotation, a beam collimation of 80 × 0.5 mm and a pitch factor of 0.813. The tube current modulation system was disabled, and tube current values were set (90/180/360 mA) to obtain volume CT dose indexes (CTDI_vol_) of 12.7, 5.9 and 3.0 mGy. These three dose levels corresponded to the dose reference level used in our country, the dose level used at our institution for CT examinations of the abdomen, and the low-dose levels used at our institution for the abdomen [29]. Ten CT acquisitions were performed for each dose level.

Raw data were reconstructed using the three available levels (level-1, level-2, level-3) of the AiCE and PIQE algorithms. The only 512^2^ pixel matrix available with AiCE on this CT system was used, and a 1,024^2^ pixel matrix for PIQE was chosen for the super-resolution reconstruction mode. The Body reconstruction kernel available for both algorithms and used in clinical practice for abdominal CT images was used. A slice thickness of 1 mm (1 mm increment) and a 320.3 mm field of view were used for all reconstructed images. Beam Hardening Correction was activated for each reconstruction.

### Task-based image quality assessment

A task-based image quality assessment was performed using the iQMetrix-CT software v1.2 [30]. For each dose level and each DLR algorithm, the NPS and TTF were computed on all the data from the 10 acquisitions.

#### Noise power spectrum

For each DLR algorithm and each dose level, the NPS was computed in 50 axial slices (5 consecutive slices for each of the 10 acquisitions) by placing eight square regions of interest (ROIs) of 104^2^ pixels (Fig. 1a), as follows:

1 $${{NPS}}_{2D}\left({f}_{x},{f}_{y}\right)=\frac{{\Delta }_{x}{\Delta }_{y}}{{L}_{x}{L}_{y}}\frac{1}{{N}_{{ROI}}}{\sum }_{i=1}^{{N}_{{ROI}}}{\left|{{FFT}}_{2D}\left\{{{ROI}}_{i}\left(x,y\right)-{{FIT}}_{i}(x,y)\right\}\right|}^{2}$$

wherein *Δ*_*x*_ and *Δ*_*y*_ are the pixel size in the *x*- and *y*-directions, respectively; *L*_*x*_ and *L*_*y*_ are the lengths of the ROIs in the *x*- and *y*-directions; *N*_*ROI*_ is the number of ROIs; *FFT* is the Fast Fourier transform; ${{ROI}}_{i}\left(x,y\right)$ is the mean pixel value of a ROI measured at its position (x,y) and ${{FIT}}_{i}(x,y)$ is a 2^nd^ order polynomial fit of ${{ROI}}_{i}\left(x,y\right)$.

To quantify the changes in noise magnitude, the square root of the area under the NPS2D curve (HU) and the magnitude of the NPS1D peak (HU².mm²) were measured. The average spatial frequency (f_av_, mm^-1^) of the NPS1D curve was used to quantify changes in noise texture [31]. In practical terms, noise magnitude reflects how ‘grainy’ the image appears, while the average spatial frequency (f_av_) describes whether the noise texture is fine- or coarse-grained.

#### Task-based transfer function

For each dose level, the TTF was computed on the Solid Water^®^ and iodine at 10 mg/mL inserts using the circular edge technique (Fig. 1b) [32]. To minimize the image-noise effect, the TTF was computed from 100 consecutive axial slices (10 slices for each of the 10 acquisitions) [31]. The TTF values at 50% (f_50_, mm^-1^) were used to quantify changes in spatial resolution. The f_50_ value represents the sharpness of object edges: higher values indicate sharper, more distinct boundaries between tissues.

#### Detectability index

Detectability indexes are used to estimate the radiologist’s ability to perform a clinical task. It corresponds to a figure of merit reflecting the resolution and noise properties (TTF and NPS outcomes) as they relate to the ability of the system to perform a task of interest. Different model observer model can be used to calculate the detectability index. Among the most commonly used are the non-prewhitening observer model with an eye filter (NPWE). This model observer incorporates a model of the human visual system and its non-uniform response to different spatial frequencies and it was computed as follows:

2 $${d}_{{NPWE}}^{{\prime} 2}=\frac{{\left[\iint {{|W}(u,v)|}^{2}\times {TTF}{\left(u,v\right)}^{2}\times E{\left(u,v\right)}^{2}{dudv}\right]}^{2}}{\iint {{|W}\left(u,v\right)|}^{2}\times {TTF}{\left(u,v\right)}^{2}\times {NPS}(u,v)\times {E(u,v)}^{4}{dudv}}$$

wherein $u$ and $v$ are the spatial frequencies in the x- and y-directions, $E$ the eye filter that models the human visual system sensitivity to different spatial frequencies [33], and $W\left(u,v\right)$ the task function defined as:

3 $$W=\left|{{FFT}}_{2D}\left\{{h}_{1}\left(x,y\right)-{h}_{0}\left(x,y\right)\right\}\right|$$

wherein ${FFT}$ is the Fast Fourier Transform, ${h}_{1}\left(x,y\right)$ and ${h}_{0}\left(x,y\right)$ are the object present and object absent hypotheses, respectively [31, 34].

The detectability index (d′) is a model-based estimate of how easily an observer could detect a lesion. Higher values imply better modeled lesion visibility. The detectability indexes (d’) of two 10-mm diameter clinical tasks approaching the contrast of a contrast-enhanced high-contrast task (*e.g*., enhanced vascular or strongly enhancing parenchymal structure) and an unenhanced low-contrast task (*e.g*., non-vascular lesions or hematoma) were calculated [29]. The TTF outcomes of the iodine insert and a contrast of 350 HU were used for the first task and the TTF outcomes of the Solid Water^®^ insert and a contrast of 85 HU for the second task.

The shape signal was circular, and the contrast profile was “Gaussian” [31]. The interpretation conditions for calculating d’ were a display size of 180 mm, a 500 mm viewing distance, and the Eckstein visual function [33, 35].

## Results

### Noise power spectrum

**Fig. 2:**
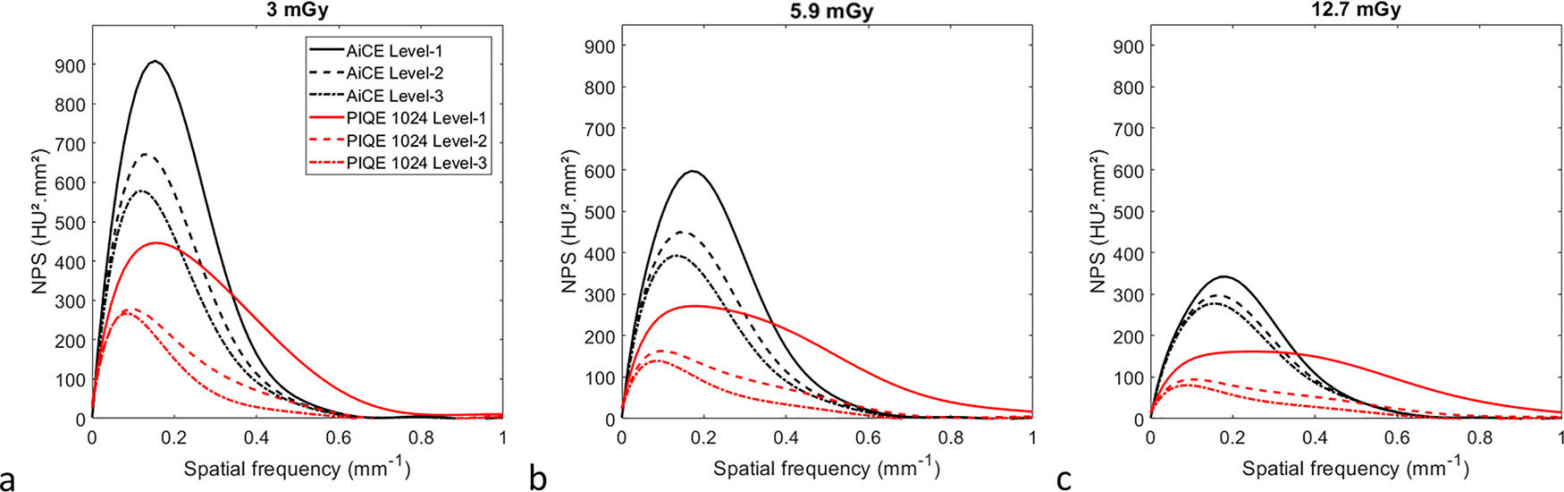
Noise power spectrum curves (NPS) obtained for the three levels of the AiCE and PIQE algorithms at 3.0 (**a**), 5.9 (**b**), and 12.7 (**c**) mGy

Figure 2 depicts the 1D NPS curves obtained for the three dose levels and the three levels available on the two DLR algorithms.

#### Noise magnitude

For both DLR algorithms, noise magnitude and NPS1D peak values decreased as the dose levels and DLR levels increased (Table 1). For AiCE and all dose levels, noise magnitude values decreased by -12.9 ± 5.4% on average from level-1 to level-2 and by -7.2 ± 3.3% from level-2 to level-3. For PIQE, this decrease was greater, with -44.4 ± 0.8% and -27.4 ± 3.8%, respectively. For all DLR and dose levels, NPS1D peaks were lower with PIQE than with AiCE, with a mean reduction of -59.8 ± 7.3%. For level-1, noise magnitude values were higher with PIQE than with AiCE, and these differences increased with the dose level. Noise magnitude values were lower with PIQE than with AiCE for level-2 (-27.6 ± 3.8%) and level-3 (-43.5 ± 1.4%).

**Table 1:** Values of noise magnitude and average noise power spectrum (NPS) spatial frequency (f_av_) obtained for each dose level and with each level (level-1, level-2, level-3) of the AiCE and PIQE algorithms

	CTDI_vol_ (mGy)	AiCE 512^2^			PIQE 1,024^2^		
		Level-1	Level-2	Level-3	Level-1	Level-2	Level-3
NPS1D peak (HU².mm²)	3.0	908.7	671.2	578.9	446.2	278.0	266.1
	5.9	596.9	449.8	393.0	271.2	163.0	139
	12.7	342.1	296.9	277.6	161.7	94.5	80.4
Noise magnitude (HU)	3.0	17.8	14.9	13.5	17.9	10.1	7.8
	5.9	16.1	13.5	12.3	18.2	10.0	7.0
	12.7	12.5	11.7	11.3	16.0	8.8	6.2
f_av_ (mm^-1^)	3.0	0.20	0.19	0.19	0.28	0.20	0.16
	5.9	0.22	0.22	0.21	0.35	0.26	0.20
	12.7	0.23	0.23	0.23	0.40	0.30	0.24

*CTDI*_*vol*_ Volume CT dose index

#### Noise texture

For both DLR algorithms and at all dose levels, the NPS curves shifted toward lower frequencies as the DLR levels increased, and these shifts were more pronounced for PIQE than for AiCE (Fig. 2 and Table 1). For all dose levels, the mean differences in average NPS spatial frequency (f_av_) were -25.2 ± 1.8% from level-1 to level-2 and -22.0 ± 0.9% from level-2 to level-3 for PIQE and for AiCE -2.8 ± 1.7% and -1.5 ± 1.3%, respectively. For all DLR levels, f_av_ values decreased as the dose levels increased (Table 1). For AiCE and all DLR levels, f_av_ values increased by 10.9 ± 0.5% on average from 3 to 5.9 mGy and 6.2 ± 3.0% from 6 to 12.7 mGy. For PIQE and all DLR levels, the increase was greater with 27.4 ± 1.4% and 14.7 ± 2.6%, respectively. Compared to AiCE, f_av_ values were higher with PIQE for all dose levels for level-1 (55.9 ± 16.7%) and level-2 (20.1 ± 13.9%), and the opposite pattern was found for level-3, except at 12.7 mGy.

### Task-based transfer function

**Fig. 3:**
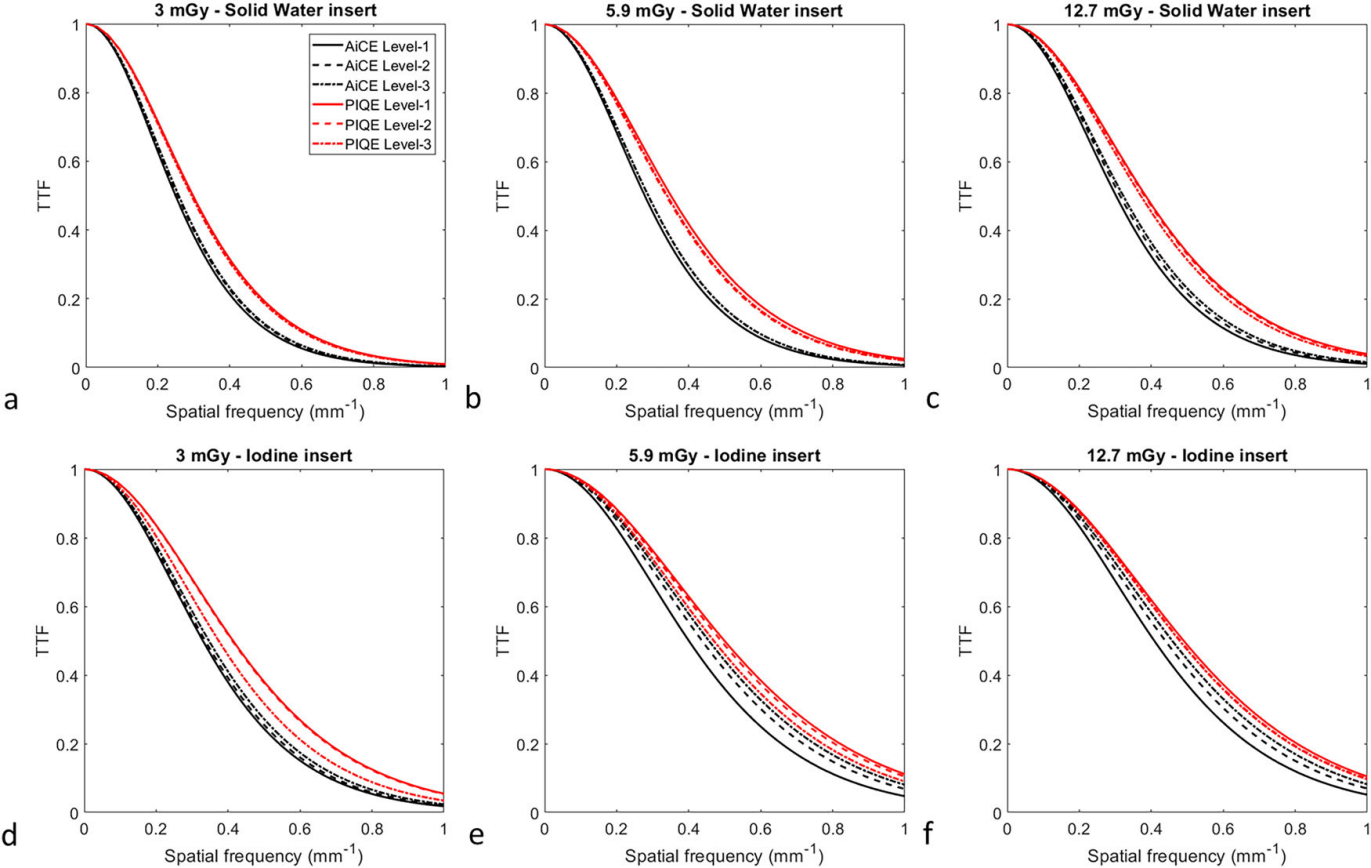
Task-based transfer function curves of Solid Water^®^ (**a**–**c**) and 10 mg/mL iodine inserts (**d**–**f**) obtained for the three levels of the AiCE and PIQE algorithms at 3.0 (**a**, **d**), 5.9 (**b**, **e**) and 12.7 (**c**, **f**) mGy

For both inserts, both DLR algorithms and all DLR levels, the TTF curves shifted toward higher frequencies as the dose levels increased (Fig. 3).

For the Solid Water^®^ insert and at each dose level (Table 2), similar f_50_ values were found between DLR levels for AiCE algorithm (0.254 ± 0.005 mm^-1^ at 3 mGy, 0.285 ± 0.006 mm^-1^ at 5.9 mGy and 0.313 ± 0.010 mm^-1^ at 12.7 mGy). A similar pattern was found with the PIQE algorithm, and f_50_ values were higher with PIQE than with AiCE for each dose level (0.295 ± 0.003 mm^-1^ at 3 mGy, 0.343 ± 0.007 mm^-1^ at 5.9 mGy and 0.379 ± 0.006 mm^-1^ at 12.7 mGy).

**Table 2:** Values of task-based transfer function at 50% (f_50_) for Solid Water^®^ and iodine at 10 mg/mL inserts obtained for each dose level and at each level (level-1, level-2, level-3) of the AiCE and PIQE algorithms

	CTDI_vol_ (mGy)	AiCE 512^2^			PIQE 1,024^2^		
		Level-1	Level-2	Level-3	Level-1	Level-2	Level-3
f_50_ (mm^-1^) Solid Water^®^	3.0	0.25	0.26	0.26	0.30	0.29	0.29
	5.9	0.28	0.29	0.29	0.35	0.34	0.34
	12.7	0.30	0.31	0.32	0.38	0.38	0.37
f_50_ (mm^-1^) Iodine	3.0	0.33	0.34	0.35	0.41	0.41	0.37
	5.9	0.40	0.44	0.46	0.49	0.48	0.47
	12.7	0.41	0.44	0.46	0.50	0.49	0.48

*CTDI*_*vol*_ Volume CT dose index

For the iodine insert and at all dose levels, f_50_ values increased as the DLR level increased with AiCE (6.2 ± 3.9% from level-1 to level-2 and 3.9 ± 0.8% from level-2 to level-3). The opposite pattern was found with PIQE (-1.4 ± 0.8% from level-1 to level-2 and -4.7 ± 4.6% from level-2 to level-3). f_50_ values were higher with PIQE than with AiCE for all dose levels and DLR levels, and the differences between the two DLR algorithms decreased as the dose level increased (23.9 ± 3.2% for level-1 and 10.5 ± 6.2% for level-3).

### Detectability indexes

**Fig. 4:**
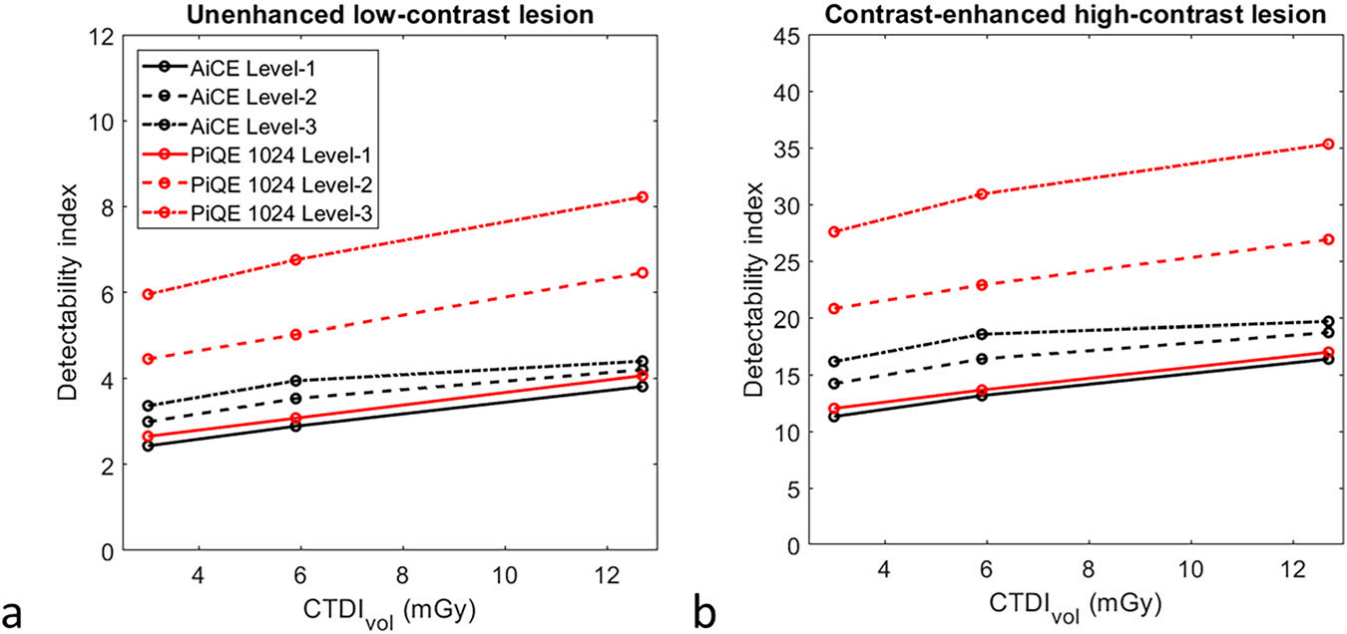
Detectability index (d’) values for the unenhanced low-contrast lesion (**a**) and contrast-enhanced high-contrast lesion (**b**) obtained for the three levels of the AiCE and PIQE algorithms

For both DLR algorithms and both simulated lesions, d’ values increased as the dose level and the DLR level increased (Fig. 4 and Table 3). The increase in d’ values according to DLR level was greater for PIQE (64.7 ± 5.8% from level-1 to level-2 and 32.4 ± 4.8% from level-2 to level-3) than for AiCE (20.0 ± 6.3% and 10.2 ± 4.1%, respectively).

**Table 3:** Values of the detectability index (d’) for unenhanced low-contrast and contrast-enhanced high-contrast simulated lesions obtained for each dose level and at each level (level-1, level-2, level-3) of the AiCE and PIQE algorithms

	CTDI_vol_ (mGy)	AiCE 512^2^			PIQE 1,024^2^		
		Level-1	Level-2	Level-3	Level-1	Level-2	Level-3
d’ ‒ Unenhanced low-contrast lesion	3.0	2.4	3.0	3.4	2.7	4.5	6.0
	5.9	2.9	3.5	4.0	3.1	5.0	6.8
	12.7	3.8	4.2	4.4	4.1	6.5	8.2
d’ ‒ Contrast-enhanced high-contrast lesion	3.0	11.3	14.2	16.2	12.0	20.8	27.6
	5.9	13.1	16.4	18.6	13.6	22.9	30.9
	12.7	16.4	18.7	19.7	17.0	26.9	35.3

*CTDI*_*vol*_ Volume CT dose index

For both simulated lesions and at all DLR levels, d’ values were higher with PIQE than with AiCE. The differences between AiCE and PIQE were more marked for level-2 (45.7 ± 5.0%) and level-3 (75.2 ± 7.3%). At level-1, d’ values were higher by 7.4 ± 1.5% on average for the unenhanced low-contrast lesion (Fig. 4a) and 4.6 ± 1.5% for the contrast-enhanced high-contrast lesion (Fig. 4b).

## Discussion

In the present study, the impact of a new SR-DLR algorithm, PIQE, for improving abdominal CT image quality was assessed. To do this, a task-based image quality assessment was performed, and the results obtained with PIQE were compared with those obtained with an NR-DLR algorithm, AiCE, commonly used in clinical practice. Compared to AiCE, PIQE improved the spatial resolution and detectability of simulated low and high-contrast abdominal lesions. Compared to AiCE, the image noise reduction and improvements in image texture with PIQE depended on the DLR levels. These outcomes represent descriptive performance measures obtained under controlled phantom conditions. Because only a single measurement was generated per condition, no statistical inference was possible.

The NPS results confirmed that the noise magnitude decreased as the dose level and the DLR level increased for both DLR algorithms. At the same dose and DLR levels, the magnitude of the NPS1D curves was lower with PIQE than with AiCE. This resulted in lower noise magnitude values with PIQE compared to AiCE for all levels, except for level-1. For this level, although the one-dimensional NPS curves with PIQE had a lower magnitude than with AiCE, they extended over a wider range of spatial frequencies, leading to an increase in the noise magnitude values for the three dose levels assessed. We also found that the reductions in noise magnitude values with increasing DLR levels were more marked with PIQE than with AiCE, particularly from level-1 to level-2. In terms of noise texture, the NPS1D curve values shifted toward low spatial frequencies as the dose level decreased and DLR levels increased for both DLR algorithms. These changes in f_av_ values according to dose and DLR levels were more marked for PIQE than for AiCE. Compared to AiCE, f_av_ values were higher with PIQE for all dose levels but only for level-1 and level-2. Visually, the shift of the NPS1D curves toward low spatial frequencies and, therefore, the decrease in f_av_ values, resulted in a smoother image and coarser granularity. The same variations in noise magnitude and noise texture values according to dose and DLR levels were found for AiCE with the same reconstruction kernel [36] and with PIQE, but with the cardiac kernel and a matrix size of 512^2^ pixels [22].

The TTF results obtained confirmed that the spatial resolution depended both on the contrast of the inserts used and also on the dose level related to the non-linear properties of the two DLR algorithms used [6]. For both inserts, the values of TTF at 50% (f_50_) shifted toward higher frequencies as the dose level increased and were higher for the high-contrast iodine insert than for the low-contrast Solid Water^®^ insert at all doses and DLR levels. For the low-contrast insert, the values of f_50_ were slightly influenced by the DLR level for both algorithms. For the high-contrast insert, the DLR level had an impact on the spatial resolution. For AiCE, the f_50_ values increased with the increase in the DLR level, while the opposite was true for PIQE. These variations in spatial resolution depending on the contrast of the insert were also found in the literature for AiCE [36]. In all cases, regardless of the insert used, the f_50_ values were higher with PIQE than with AiCE at the same dose level and the same DLR level. Similar results were found for both PIQE and AiCE but with a matrix size of 512^2^ pixels and the Cardiac kernel [22]. Visually, this would be reflected in the images by sharper contours of structures and would make it easier to distinguish between similar structures. This improvement in terms of spatial resolution could be explained by a smaller pixel size for PIQE in super-resolution reconstruction mode and the training of the PIQE CNN from an ultra-high-resolution CT.

The results of the detectability index confirmed that the d’ values increased as the dose level and the DLR level increased for both algorithms. The same pattern was found with AiCE [36] and PIQE [22] for the different conditions defined previously (different phantom and/or dose level and/or matrix size and/or kernel). We also found that the d’ values were higher with PIQE than with AiCE for the two simulated lesions and for all DLR levels. The improvements in d’ values were more marked with level-2 and level-3 than with level-1. As the contrast of the simulated lesions was fixed, these results were directly related to variations in TTF and NPS outcomes. With level-1, the noise magnitude was higher with PIQE than with AiCE, but this increase was compensated by higher f_50_ values for both inserts and higher f_av_ values as well. For the other two DLR levels, all metrics were better with PIQE than with AiCE.

Finally, these combined results open up interesting prospects for the use of PIQE in super-resolution reconstruction mode in clinical routine to replace AiCE in normal-resolution reconstruction mode for the detection of abdominal lesions. To reduce the image noise and improve the detectability of lesions whilst improving image texture and spatial resolution, the use of level-2 seems to be the most appropriate. These initial *in vivo* results have good clinical prospects, particularly for helping radiologists make the right diagnosis with abdominal lesions. These results also pave the way for optimizing the doses delivered to patients with PIQE in super-resolution reconstruction mode rather than AiCE for abdominal CT scans. Although AiCE has already made it possible to reduce doses considerably, PIQE has even greater potential for reducing doses in clinical routine. However, although these preliminary results on phantoms are encouraging, they cannot be directly used in routine clinical practice. Commonly used image quality phantoms do not take into account anatomical complexity, movement, and contrast variability in clinical task detection. These differences could impact the results of noise magnitude and texture, spatial resolution, and even detectability. The results obtained in this study must now be validated for routine abdominal CT with a cohort of patients for a wide range of abdominal lesions.

This study has its limitations. Acquisitions were made on only one phantom, but this phantom does not take into account the various morphologies of patients undergoing abdominal CT examination. Besides, the inserts used to simulate lesions in the phantom do not represent exactly the same features as the anatomical structures of patients. Then, we chose to compare AiCE with PIQE with different pixel matrix sizes because we wanted to evaluate the latest version of PIQE under maximum resolution conditions. The results obtained with PIQE with a matrix size of 512^2^ pixels would probably be different, particularly in terms of spatial resolution, noise magnitude and noise texture. However, PIQE in super-resolution reconstruction mode was developed for use in clinical routine as a replacement for AiCE. In clinical practice, improvements in d′ values observed here may not directly translate into proportional gains in lesion detection, since observer performance is also influenced by anatomical background, patient motion, and lesion variability. Validation in studies involving human readers and patient cohorts will be required to determine the true diagnostic impact of SR-DLR. Last, no statistical analysis was performed because only one calculation of NPS, TTF per insert, and d’ value per simulated lesion was performed per dose level and per type of reconstruction.

In conclusion, compared to AiCE in normal-resolution reconstruction mode, PIQE in super-resolution reconstruction mode improved spatial resolution and detectability of simulated low and high-contrast abdominal lesions. The best combined results in terms of noise reduction and improvement of noise texture, spatial resolution and detectability of simulated abdominal lesions were obtained with level-2 of SR-DLR. Using PIQE in super-resolution reconstruction mode therefore presents numerous prospects for improving abdominal CT images and a high potential for reducing the doses delivered to patients. These results, while encouraging, are exploratory and limited to a single phantom. Further validation in clinical cohorts will be essential before routine adoption.

## Data Availability

The datasets analyzed during the current study are available from the corresponding author on reasonable request.

## Abbreviations

AiCE: Advanced intelligent Clear-IQ Engine
CT: Computed tomography
d’: Detectability index
DLR: Deep-learning reconstruction
NPS: Noise power spectrum
NR-DLR: Normal-resolution deep learning reconstruction
PIQE: Precise IQ Engine
SR-DLR: Super-resolution deep learning reconstruction
TTF: Task-based transfer function
